# Methylome-wide Association Study of Atrial Fibrillation in Framingham Heart Study

**DOI:** 10.1038/srep40377

**Published:** 2017-01-09

**Authors:** Honghuang Lin, Xiaoyan Yin, Zhijun Xie, Kathryn L. Lunetta, Steven A. Lubitz, Martin G. Larson, Darae Ko, Jared W. Magnani, Michael M. Mendelson, Chunyu Liu, David D. McManus, Daniel Levy, Patrick T. Ellinor, Emelia J. Benjamin

**Affiliations:** 1 National Heart Lung and Blood Institute’s and Boston University’s Framingham Heart Study, Framingham, MA, USA; 2 Section of Computational Biomedicine, Department of Medicine, Boston University School of Medicine, Boston, MA, USA; 3 TCM Clinical Basis Institute, Zhejiang Chinese Medicine University, Hangzhou, Zhejiang, China; 4 Department of Biostatistics, Boston University School of Public Health, Boston, MA, USA; 5 Cardiovascular Research Center, Massachusetts General Hospital, Charlestown, MA, USA; 6 The Broad Institute of Harvard and MIT, Cambridge, MA, USA; 7 Department of Mathematics and Statistics, Boston University, Boston, MA, USA; 8 Division of Cardiology, Department of Medicine, UPMC Heart and Vascular Institute, University of Pittsburgh, Pittsburgh, PA, USA; 9 Section of Cardiovascular Medicine, Department of Medicine, Boston University School of Medicine, Boston, MA, USA; 10 Population Sciences Branch, Division of Intramural Research, National Heart, Lung, and Blood Institute, Bethesda, MD, USA; 11 Department of Cardiology, Boston Children’s Hospital, Boston, MA, USA; 12 Medicine and Quantitative Health Sciences, University of Massachusetts Medical School, Worcester, MA, USA; 13 Section of Preventive Medicine, Department of Medicine, Boston University School of Medicine, Boston, MA, USA; 14 Department of Epidemiology, Boston University School of Public Health, Boston, MA, USA.

## Abstract

Atrial fibrillation (AF) is the most common cardiac arrhythmia, but little is known about the molecular mechanisms associated with AF arrhythmogenesis. DNA methylation is an important epigenetic mechanism that regulates gene expression and downstream biological processes. We hypothesize that DNA methylation might play an important role in the susceptibility to develop AF. A total of 2,639 participants from the Offspring Cohort of Framingham Heart Study were enrolled in the current study. These participants included 183 participants with prevalent AF and 220 with incident AF during up to 9 years follow up. Genome-wide methylation was profiled using the Illumina Infinium HumanMethylation450 BeadChip on blood-derived DNA collected during the eighth examination cycle (2005–2008). Two CpG sites were significantly associated with prevalent AF, and five CpGs were associated with incident AF after correction for multiple testing (FDR < 0.05). Fourteen previously reported genome-wide significant AF-related SNP were each associated with at least one CpG site; the most significant association was rs6490029 at the *CUX2* locus and cg10833066 (*P* = 9.5 × 10^−279^). In summary, we performed genome-wide methylation profiling in a community-based cohort and identified seven methylation signatures associated with AF. Our study suggests that DNA methylation might play an important role in AF arrhythmogenesis.

More than 30 million people worldwide are currently affected by atrial fibrillation (AF)^1^,^2^, the most common cardiac arrhythmia. AF is associated with a significantly increased risk for stroke^3^, dementia^4^, heart failure^5^,^6^,^7^, myocardial infarction^8^, and death^9^,^10^,^11^. In the past few years, genome-wide association studies (GWAS) have identified 14 genetic loci associated with AF^12^,^13^,^14^,^15^. However, none of these loci is located in protein coding regions, and the molecular mechanisms underlying the associations remain largely unknown.

DNA methylation refers to the process of adding a methyl group to the cytosine of cytosine-phosphate-guanine dinucleotides (CpG). DNA methylation stabilizes chromatin structure during transcription, which can regulate many downstream transcriptional processes. The methylation state can be transmitted through cell division, and can vary across multiple tissues^16^ or over an individual’s lifetime. Recent studies found that alteration of DNA methylation is linked to many cardiovascular disease-related disorders, such as atherosclerosis^17^,^18^,^19^,^20^, high blood pressure^21^,^22^, and diabetes^23^,^24^,^25^. Several CVD risk factors, including smoking^26^, obesity^27^, and alcohol consumption^28^, are also associated with DNA methylation changes. However, there are no previous studies examining the relation of DNA methylation with prevalent or incident AF among community-based adults.

We hypothesized that DNA methylation might contribute to AF susceptibility. We performed a large-scale DNA methylation profiling study in participants from the Framingham Heart Study, and examined the association of DNA methylation with prevalent and incident AF. Given the importance of DNA methylation to gene expression, we investigated the association of DNA methylation signatures of AF with gene expression from the same group of participants. We also assessed the association of GWAS loci for AF with DNA methylation to examine potential implications of AF-related genetic variants on DNA methylation.

## Results

### Differential Methylation

Table 1 shows the descriptive characteristics of 2,639 eligible participants (mean age 65 ± 9 years, 57% women), including 183 participants who developed AF before the eighth examination (prevalent AF), and an additional 220 participants who developed AF after the examination (incident AF) through follow-up in 2014.

**Figure 1:**
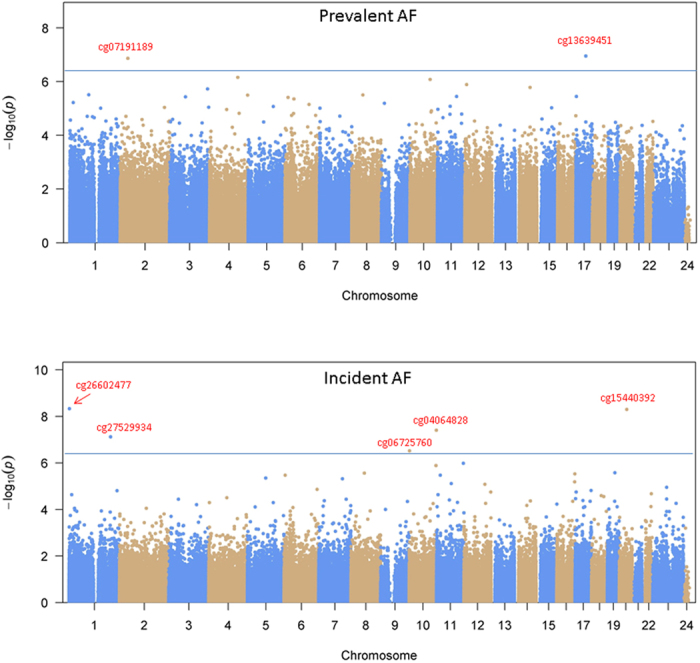
Manhattan plot of CpG sites associated with prevalent and incident AF. The x-axis represents the chromosome, and the y-axis represents the log_10_(p-value) of the associations with prevalent and incident AF. The horizontal line represents the significance cutoff (FDR = 0.05). We marked CpGs that were significantly associated with prevalent or incident AF (FDR < 0.05).

Figure 1 shows the Manhattan plots of CpG sites associated with prevalent and incident AF. As shown in Table 2, two CpG were significantly associated with prevalent AF (FDR < 0.05). One significant CpG site was cg13639451 (*P* = 1.1 × 10^−7^, FDR = 0.03), located 1447 bp upstream of *WFIKKN2*, which encodes an endopeptidase inhibitor with a known role in muscle fiber development via inhibition of myostatin^29^. The other one was cg07191189 (*P* = 1.4 × 10^−7^, FDR = 0.03), located 75 bp upstream of *STRN* (*Striatin*). The CpG site also was predicted to affect the binding of transcription factor TFII-I^30^. Five CpG sites were associated with incident AF (FDR < 0.05). The most significant CpG site was cg26602477 (*P* = 4.7 × 10^−9^), located 207 bp downstream of *SSU72*. The results were similar when we additionally adjusted for different cell counts (Supplemental Table 1).

In our secondary analysis, we tested the associations of the seven significant prevalent or incident AF associated CpG sites adjusting for selected AF risk factors^31^, including smoking, height, weight, systolic blood pressure, diastolic blood pressure, prevalent diabetes mellitus, prevalent myocardial infarction, prevalent heart failure, and antihypertensive treatment. As shown in Supplemental Table 1, the results were similar to the primary analysis.

We also tested the association of methylation with all AF cases (combining prevalent and incident AF), but none of CpG site was significant after correction for multiple testing. The top CpG sites for all AF are listed in Supplemental Table 2.

**Figure 2:**
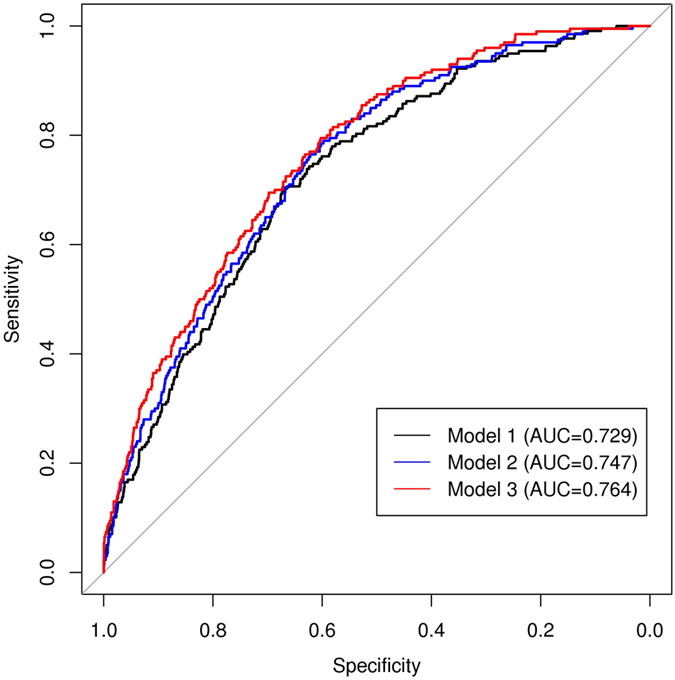
Receiver operating characteristic (ROC) curves of three models to predict incident AF. Model 1: Only included traditional risk factors; Model 2: Included traditional risk factors and 14 AF-related genetic loci; Model 3: Included traditional risk factors, 14 AF-related genetic loci, and 5 AF-related CpG sites. The inclusion of genetic loci and methylation profiles modestly improved the prediction performance with area under curve (AUC) increasing from 0.729 (model 1) to 0.747 (model 2) and 0.764 (model 3).

We then examined if the inclusion of methylation profiles would improve the prediction of AF compared to traditional risk factors. Three models were tested: Model 1: Only included traditional risk factors associated with incident AF^31^; Model 2: Included traditional risk factors and 14 published AF-related genetic loci^12^,^13^,^14^,^15^; Model 3: Included traditional risk factors, 14 known genetic loci associated with AF, and 5 CpG sites associated with incident AF. As shown in Fig. 2, the inclusion of genetic loci and methylation profiles modestly improved the prediction performance with area under curve (AUC) increasing from 0.729 (model 1) to 0.747 (model 2) and 0.764 (model 3).

### DNA Methylation Associated with Gene Expression

DNA methylation is an important mechanism to regulate gene expression. We tested if any of the seven AF-related CpG sites was associated with gene expression or not. We stratified our analysis by *cis*-gene associations (defined as those within 1 Mb of the CpG site), and *trans*-genes (defined as those more than 1 Mb away from the CpG site or in different chromosomes). A total of 175 *cis-*associations and 124, 936 *trans-*associations were found and tested. Therefore the significance cutoff was 0.05/175 = 2.9 × 10^−4^ for *cis*-associations and 0.05/124,936 = 4.0 × 10^−7^ for *trans*-associations. The most significant *cis*- and *trans*- genes for each CpG site are shown in Table 3.

None of the *cis*-associations reached significance after adjusting for multiple testing (all with *P* > 2.9 × 10^−4^). Two AF-related CpG sites (cg13639451 and cg15440392) were associated with the expression *trans*-genes (*P* < 4.0 × 10^−7^). cg13639451 was associated with the expression of *RPS18* (*P* = 4.5 × 10^−20^), and cg15440392 was associated with the expression of *GZMH* (*P* = 7.0 × 10^−9^).

### DNA Methylation Associated with AF-related Genetic Loci

**Figure 3:**
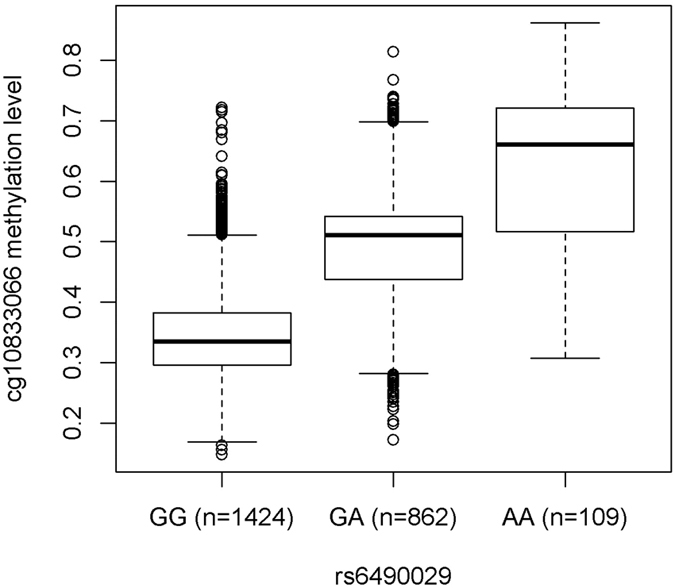
Association of SNP rs6490029 (*CUX2*) with the methylation of cg10833066. The boxplot indicates the minimum, 25%, 50%, 75% and the maximum methylation level for each genotype. Outliers were marked as points. Samples with one or two “A” alleles at rs6490029 tended to have a higher methylation level. The number of samples with each genotype was marked as well.

DNA methylation may be regulated by both genetic and environmental factors. Fourteen AF-related SNPs have been previously reported^12^,^13^,^14^,^15^, however, their association with nearby DNA methylation has not been studied yet. For each AF-related SNP, we studied its association with the methylation of CpG sites within 1 Mb. A total of 6042 SNP-CpG pairs were identified and tested; therefore the significance cutoff was defined as *P* < 0.05/6042 = 8.3 × 10^−6^. As shown in Table 4, all the AF-related SNPs were significantly associated with methylation of at least one CpG site. The most significant association was between rs6490029 at the *CUX2* locus and cg10833066 (*P* = 9.5 × 10^−279^). As shown in Fig. 3, the methylation level of cg10833066 increased with increasing copies of the “A” allele of rs6490029, which also was associated with higher AF risk according to previous GWAS^13^.

## Discussion

DNA methylation is an important process regulating gene expression without changing DNA sequence. We performed genome-wide methylation profiling of more than 2600 participants from the Framingham Heart Study Offspring cohort. Two CpG sites were found to be significantly associated with prevalent AF, and five other CpG sites were associated with incident AF. We also examined the association between methylation and GWAS loci for AF, and found that all AF loci were associated with the methylation of at least one CpG site, suggesting DNA methylation might be a possible mechanism through which AF-specific genetic variations affect gene regulation.

Interestingly, the most significant CpG site for prevalent AF (cg13639451) was also associated with the expression of the ribosomal protein *RPS18* (*P* = 4.5 × 10^−20^). The gene has been shown to bind to *CAMK2D*^32^, a gene encoding a calcium/calmodulin-dependent protein kinase II that plays an important role in excitation-contraction coupling in the heart^33^. The other significant CpG site for prevalent AF, cg07191189, was located 75 bp upstream of *STRN*, which encodes striatin, a calmodulin-dependent scaffolding protein. Striatin has been shown to bind directly to caveolin-1^34^, encoded by *CAV1*, which is involved in cardiac development^35^. Further, the *CAV1* locus was associated with AF in our previous GWAS^12^. Given that all known GWAS loci for AF are located in intergenic or intronic regions, our results suggest that methylation might act as an important bridge to link genetic variation and disease susceptibility.

The most significant CpG site for incident AF was cg26602477, located 207 bp away from *SSU7*, a gene encoding a protein phosphatase that regulates the dephosphorylation process of RNA polymerase^36^. Another significant CpG site cg15440392 is located within *BLCAP*, which encodes a bladder cancer-associated protein. The gene is highly conserved through evolution and is expressed in a variety of human tissues including cardiac^37^. However, the implications of these genes for AF risk are still unknown.

The AF-related SNP rs6490029 was significantly associated with CpG site cg10833066, which is located ~400 kb upstream of *ALDH2*, a gene encoding aldehyde dehydrogenase. Aldehyde dehydrogenase plays an important role in the metabolism of alcohol through catalyzing the oxidation of aldehydes into carboxylic acids. Previous studies have proven that excessive alcohol usage is associated with increased risk of AF^38^,^39^,^40^, suggesting that DNA methylation might be an important factor in AF susceptibility.

It is worth noting that there is no overlap between CpG sites that were associated with prevalent and incident AF. Several reasons might be responsible for the lack of overlap. Unlike genetic variations, the relations between DNA methylation and AF are reciprocal^41^, which means that not only DNA methylation could affect the susceptibility of AF, the disease could also influence one’s methylation profile. Given that DNA methylation was profiled from whole blood collected during a routine examination, the methylation profile in samples with prevalent AF could have been influenced by AF for an extended period of time. In contrast, for samples with incident AF, the methylation profile could be one of factors that influenced their susceptibility to develop AF. In addition, there was a slight difference between the numbers of incident AF cases compared to prevalent AF cases, which might also have contributed to the variation in the methylation profile identified.

We acknowledge several limitations in our study. DNA methylation was collected at a single examination, so we were unable to investigate longitudinal changes in methylation profiles. Moreover, the DNA methylation profile was measured from whole blood, which could vary from levels in atria or specific white blood cell types. However, invasive specimen collection is not feasible in a community-based study. The participants in our study are largely middle-age to older and are of European decent, so it is unclear whether our findings are generalizable to other ages or races/ethnicities. As an observational study we cannot exclude residual confounding, or determine causal relationships. Given that many other cohorts have very limited numbers of AF cases with methylation profiling, and they are usually derived from different types of cells, we were unable to replicate our findings in the current study. Future studies with multiple examinations and population diversity might provide better understanding of methylation signatures for AF and are required to verify our findings.

In conclusion, we examined the association of DNA methylation with AF in a moderately large community-based cohort, and identified multiple methylation signatures associated with AF. Our results suggest that DNA methylation might represent an important bridge to link genetic variations with AF susceptibility. Future validation might uncover AF-specific methylation regulation mechanisms, and potentially lead to the identification of novel therapeutic targets for better treatment of AF.

## Materials and Methods

### Study Samples

The Framingham Heart Study is a three-generation community-based cohort initiated in 1948. The Framingham Offspring cohort was recruited in 1971, and consisted of 5124 participants who are the offspring and the spouses of offspring of the Original cohort^42^. The present study was focused on Offspring cohort participants who attended the eighth examination (2005–2008). All participants gave written informed consent and the study was approved by the Institutional Review Boards of National Human Genome Research Institute and Boston University Medical Center, and all experiments were performed in accordance with relevant guidelines and regulations.

### AF Ascertainment

AF was ascertained from a combination of multiple sources. Each participant was asked about his/her cardiovascular history and a 12-lead electrocardiogram was obtained during clinic visits scheduled every 4–8 years. Additional information also was solicited during surveillance interviews biennially^43^,^44^, and from cardiovascular disease-related hospitalizations and clinician visits. At least two Framingham Heart Study cardiologists reviewed all electrocardiograms available from study visits or in- and outpatient records to adjudicate incident AF.

### DNA Methylation Quantification

The fasting peripheral whole blood was collected using Gentra Puregene Blood Kits (QIAGEN, Venlo, Netherlands) in a single examination (the eighth examination). The genomic DNA was then bisulfite-treated, amplified and hybridized to the Infinium HumanMethylation450 BeadChip (Illumina, San Diego, CA) according to the manufacturer’s standard protocols^45^. The methylation assay was performed at two centers and normalized separately, and pooled together after adjusting for batch effects between two centers.

The methylation status was represented by the β value as a continuous variable between 0 and 1 representing the proportion of methylation at each CpG site. The raw data were normalized and corrected for the background noise by “DASEN” R package^46^. We excluded probes with detection *P-*values less than 0.01. The whole probe was removed if ≥1% of samples had missing values. A sample was removed if ≤95% probes had *P*-value < 0.01. We removed probes overlapping with known genetic polymorphisms from the 1000 Genomes Project Phase 1^47^. Our analysis focused on autosomal chromosomes. A total of 443,252 CpG sites were included in the current study.

### Gene Expression Profiling

Framingham gene expression profiling has been described in detail^48^,^49^,^50^. Briefly, total RNA was isolated from fasting peripheral whole blood collected during clinic visits. RNA was then amplified and reverse transcribed into cDNA, which was hybridized to the Human Exon 1.0st Array (Affymetrix, Santa Clara, CA) according to standardized protocols. We used Robust Multi-array Average^51^ method to normalize and summarize the raw data. The gene annotations were obtained from Affymetrix NetAffx Analysis Center (version 31). Only the most reliable probe sets derived from RefSeq and GenBank records were used in this study, corresponding to 17,873 distinct transcripts^48^,^49^,^50^.

### Genetic Profiling

Genetic variation was profiled by Affymetrix 550 k Array (Affymetrix, Santa Clara, CA) as previously reported^12^. We excluded variants with call rates less than 97%, Hardy–Weinberg Equilibrium *P* -values less than 1.0 × 10^−6^, or minor allele frequencies less than 0.01. The remaining variants were imputed to HapMap r22 CEU panel by Mach (v 1.0.15)^52^.

### Statistical Analyses

Our primary analysis tested the association between methylation and AF status. For prevalent AF analysis, we hypothesized that AF status could affect methylation level. We used linear mixed effects regression models to test the association between the methylation level of each CpG with prevalent AF. We specified the methylation level as the dependent measure and the AF status at the eighth examination as the exposure. For this analysis, both incident AF cases and participants with no history of AF served as the reference group. For the incident AF analysis, we hypothesized that DNA methylation could be associated with future risk of AF. We thus used Cox proportional hazards models to relate the methylation level at each CpG to incident AF (censored at the last follow-up time or death). Participants with prevalent AF at baseline were excluded from this analysis.

Both analyses were adjusted for age, sex, assay site, and pedigree structure in Framingham. The false discovery rate (FDR)^53^ was used to correct for multiple testing, and statistical significance was claimed if the FDR was less than 0.05, corresponding to *P* < 1.4 × 10^−7^. For significant associations, we additionally adjusted for the proportion of six cell types imputed via a reference panel using the Houseman method^54^, including CD8^+^ T cells, CD4^+^ T cell, natural killer cells, B cells, monocytes and granulocytes, to account for cell count heterogeneity between study groups. In our secondary analysis, we tested the association of top CpG sites with AF adjusting for additional AF risk factors^31^, including smoking, height, weight, systolic blood pressure, diastolic blood pressure, prevalent diabetes mellitus, prevalent myocardial infarction, prevalent heart failure, and antihypertensive treatment. In an additional exploratory analysis, we combined prevalent and incident AF cases to test the association of each CpG with AF.

We also developed a combined methylation score from CpG sites significantly associated with incident AF. The score for sample i is defined as 
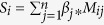
, where n is the number of CpG sites significantly associated with incident AF, *β*_*j*_ is the estimate of effect size for CpG site j, and *M*_*ij*_ is the methylation level at CpG site j for sample i. Similarly, we developed a combined genetic score from 14 top AF loci that were published previously^12^,^13^,^14^,^15^. We then combined the genetic and methylation scores together with traditional AF risk factors, and investigated their association with incident AF using Cox proportional hazards models.

### Association with Gene Expression

For each of the CpG sites associated with prevalent or incident AF, we examined the association of methylation with gene expression, stratified by *cis*-genes (defined as those within 1 Mb of the CpG site), and *trans*-genes (defined as those more than 1 Mb away from the CpG site or in different chromosomes). The association was tested by linear mixed effects regression models, whereas gene expression was treated as the dependent measure, and the methylation status was treated as the exposure, adjusted for age, sex, and family structure. We used Bonferroni correction to adjust for multiple testing, and the significance was defined as *P* < 0.05/N, where N is the number of tests.

### Association with AF-related Genetic Loci

We examined the association between genetic variations associated with AF and DNA methylation. Our analysis was limited to the 14 SNPs that were previously reported to associate with AF susceptibility by GWAS^12^,^13^. The loci included rs10821415 (*C9orf3*), rs10824026 (*SYNPO2L*), rs1152591 (*SYNE2*), rs2106261 (*ZFHX3*), rs3807989 (*CAV1*), rs3903239 (*PRRX1*), rs6666258 (*KCNN3*), rs6817105 (*PITX2*), rs7164883 (*HCN4*), rs4642101 (*CAND2*), rs13216675 (*GJA1*), rs12415501 (*NEURL*), rs10507248 (*TBX5*), and rs6490029 (*CUX2*). Linear mixed effects regression models were used to test the association between each genetic variant and DNA methylation levels of CpGs within 1 Mb of AF SNPs. Methylation levels were treated as the dependent measures and genetic variants were treated as the exposures. The analysis was adjusted for age, sex, and family structure. We used Bonferroni correction to account for multiple testing, and the significance was claimed if the *P* value of the association was less than 0.05/N, where N was determined as the number of tests.

## Additional Information

**How to cite this article**: Lin, H. *et al*. Methylome-wide Association Study of Atrial Fibrillation in Framingham Heart Study. *Sci. Rep.* **7**, 40377; doi: 10.1038/srep40377 (2017).

**Publisher's note:** Springer Nature remains neutral with regard to jurisdictional claims in published maps and institutional affiliations.

## Supplementary Material

Supplementary Materials

## Figures and Tables

**Table 1:** Clinical characteristics of studied samples.

Characteristics	No AF (n = 2,236)	Prevalent AF (n = 183)	Incident AF (n = 220)
Women, n (%)	1285 (57%)	65 (36%)	89 (40%)
Age, year ± SD	65 ± 9	72 ± 9	71 ± 8
Height, inches ± SD	66 ± 4	67 ± 4	66 ± 4
Weight, pounds ± SD	173 ± 38	190 ± 48	181 ± 43
Current smoker, n (%)	206 (9%)	8 (4%)	15 (7%)
Systolic blood pressure, mm Hg	128 ± 17	128 ± 20	135 ± 19
Diastolic blood pressure, mm Hg	74 ± 10	70 ± 10	72 ± 10
Prevalent diabetes mellitus, n (%)	316 (14%)	60 (33%)	59 (27%)
Prevalent myocardial infarction, n (%)	18 (1%)	38 (21%)	8 (4%)
Prevalent heart failure, n (%)	68 (3%)	44 (24%)	14 (6%)
Antihypertensive treatment, n (%)	1016 (45%)	130 (71%)	142 (65%)

**Table 2:** Most significant CpG sites associated with AF (FDR < 0.05).

Type	CpG site	Chr	Position+	Closest gene	Distance	Effect size	SE*	*P* value	FDR*
Prevalent AF	cg13639451	17	48911157	*WFIKKN2*	1447 bp	−0.010	0.002	1.1 × 10^−7^	0.030
	cg07191189	2	37193690	*STRN*	75 bp	0.011	0.002	1.4 × 10^−7^	0.030
Incident AF	cg26602477	1	1476845	*SSU72*	207 bp	−8.289	1.415	4.7 × 10^−9^	0.001
	cg15440392	20	36156634	*BLCAP*	301 bp	4.808	0.823	5.1 × 10^−9^	0.001
	cg04064828	10	134002751	*DPYSL4*	0	−28.669	5.218	3.9 × 10^−8^	0.006
	cg27529934	1	205054684	*RBBP5*	585 bp	−8.291	1.542	7.5 × 10^−8^	0.008
	cg06725760	10	1102461	*WDR37*	314 bp	6.655	1.300	3.1 × 10^−7^	0.027

^+^NCBI Genome Build 37.

^*^SE: Standard error; FDR: false discovery rate^53^.

**Table 3:** Association of AF-specific CpG sites with the most significant *cis*-gene expression and *trans*-gene expression.

Type	CpG site	Most significant *cis*-gene for each AF-related CpG site				Most significant *trans*-gene for each AF-related CpG site			
		Gene	Effect size	SE	*P* value	Gene^+^	Effect size	SE	P value
Prevalent AF	cg13639451	*HILS1*	0.36	0.14	9.1 × 10^−3^	*RPS18*	1.85	0.20	4.5 × 10^−20^
	cg07191189	*STRN*	0.35	0.12	4.8 × 10^−3^	*LGSN*	0.77	0.18	2.3 × 10^−5^
Incident AF	cg26602477	*C1orf159*	−0.35	0.16	3.1 × 10^−2^	*PTPN2*	−1.37	0.31	1.2 × 10^−5^
	cg15440392	*RPN2*	0.21	0.12	7.8 × 10^−2^	*GZMH*	−2.18	0.37	7.0 × 10^−9^
	cg04064828	*DPYSL4*	0.56	0.23	1.7 × 10^−2^	*ABCA4*	−0.98	0.20	7.9 × 10^−7^
	cg27529934	*PLEKHA6*	0.26	0.09	5.6 × 10^−3^	*ADAMTS2*	0.40	0.09	1.3 × 10^−5^
	cg06725760	*ZMYND11*	−0.22	0.21	2.8 × 10^−1^	*GZMH*	−3.01	0.72	3.2 × 10^−5^

^*^SE: Standard error.

^+^All the most significant genes are located in different chromosomes from the corresponding CpG sites.

^*^SE: Standard error.

**Table 4:** Most significant CpG site associated with each AF GWAS locus.

AF SNP	Cloest gene to AF SNP	CpG site	Chr	Position^+^	Closest gene to CpG site	Distance to AF SNP	Effect size	SE*	*P* value	Methylation to AF risk^$^
rs6490029	*CUX2*	cg10833066	12	111,807,467	*FAM109A*	109,010 bp	0.1380	0.0034	9.5 × 10^−279^	↑
rs10824026	*SYNPO2L*	cg02286717	10	75,415,704	*SYNPO2L*	5,504 bp	−0.0578	0.0018	1.3 × 10^−190^	↑
rs1152591	*SYNE2*	cg23250157	14	64,679,961	*SYNE2*	887 bp	−0.0109	0.0005	2.8 × 10^−97^	↓
rs7164883	*HCN4*	cg06757333	15	73,655,217	*HCN4*	3,043 bp	−0.0357	0.0019	1.3 × 10^−71^	↓
rs3807989	*CAV1*	cg12739419	7	116,140,593	*CAV2*	45,648 bp	0.0233	0.0013	1.2 × 10^−65^	↓
rs4642101	*CAND2*	cg24848339	3	12,840,334	*CAND2*	1,889 bp	−0.0112	0.0006	1.8 × 10^−63^	↑
rs12415501	*NEURL*	cg12662887	10	105,343,920	*NEURL1*	19,146 bp	0.0562	0.0034	7.5 × 10^−59^	↑
rs6666258	*KCNN3*	cg06221963	1	154,839,813	*KCNN3*	25,545 bp	0.0714	0.0052	6.6 × 10^−42^	↑
rs10507248	*TBX5*	cg10233830	12	114,701,413	*TBX5*	95,680 bp	−0.0311	0.0031	4.9 × 10^−23^	↑
rs10821415	*C9orf3*	cg13792694	9	97,865,232	*FANCC*	151,773 bp	−0.0033	0.0004	7.1 × 10^−20^	↓
rs3903239	*PRRX1*	cg09010107	1	170,638,807	*PRRX1*	69,490 bp	0.0116	0.0016	1.1 × 10^−13^	↑
rs6817105	*PITX2*	cg03587884	4	111,642,146	*PITX2*	63,622 bp	−0.0153	0.0022	1.0 × 10^−11^	↓
rs2106261	*ZFHX3*	cg06618356	16	73,097,364	*ZFHX3*	45,744 bp	−0.0096	0.0016	1.1 × 10^−9^	↓
rs13216675	*GJA1*	cg05720511	6	123,043,994	*PKIB*	591,665 bp	0.0024	0.0005	1.6 × 10^−6^	↓

All loci were significantly associated methylation status (*P* < 8.3 × 10^−6^).

^+^NCBI Genome Build 37.

^*^SE: Standard error.

^$^Indicate if AF risk allele was the same allele to increase methylation. “↑” Represents the AF risk allele would increase the methylation level, whereas “↓” represents the AF risk allele would decrease the methylation level.
