# Supplementary material for: Methylome-wide Association Study of Atrial Fibrillation in Framingham Heart Study
Source: Sci Rep. 2017 Jan 9;7:40377. doi: 10.1038/srep40377 (PMC5220313; doi:10.1038/srep40377)
Supplement: Supplementary Materials [file srep40377-s1.pdf]

Methylome-wide Association Study of Atrial Fibrillation in Framingham Heart Study

Honghuang Lin, PhD[1,2]*; Xiaoyan Yin, PhD[1]; Zhijun Xie, PhD[2,12]; Kathryn L. Lunetta, PhD[1,3]; Steven A. Lubitz, MD, MPH[4,5]; Martin G. Larson, ScD[1,8]; Darae Ko, MD[9]; Jared W. Magnani, MD, MS[1,10]; Michael M. Mendelson, MD, ScM[6,7]; Chunyu Liu, PhD[1,6]; David D. McManus, MD, ScM[11]; Daniel Levy, MD[1,6]; Patrick T. Ellinor, MD, PhD[4,5]; Emelia J. Benjamin, MD, ScM[1,10,13,14]

Table of Contents

Supplemental	Title	Page
**S. Table 1.**	Association of top CpG sites with AF (stratifying prevalent and incident AF)	**2**
**S. Table 2.**	Association of top CpG sites with both AF (combining prevalent and incident AF)	**3**

Supplemental Table 1. Association of top CpG sites with AF (stratifying prevalent and incident AF).

Type	CpG site	Chr	Position	Closest gene	Distance to gene	Cell-count Adjusted Model*			Multivariable Adjusted Model +		
						Effect size	SE$	*P* value	Effect size	SE$	*P* value
Prevalent AF	cg13639451	17	48911157	***WFIKKN2***	1447	-0.010	0.002	3.7E-07	-0.009	0.002	6.0E-06
	cg07191189	2	37193690	***STRN***	75	0.010	0.002	1.1E-06	0.013	0.002	1.5E-08
Incident AF	cg26602477	1	1476845	***SSU72***	207	-8.396	1.453	7.5E-09	-9.227	1.449	1.9E-10
	cg15440392	20	36156634	***BLCAP***	301	4.848	0.840	7.8E-09	4.476	0.882	3.9E-07
	cg04064828	10	134002751	***DPYSL4***	0	-28.007	5.168	6.0E-08	-27.965	5.155	5.8E-08
	cg27529934	1	205054684	***RBBP5***	585	-8.324	1.553	8.3E-08	-9.016	1.642	4.0E-08
	cg06725760	10	1102461	***WDR37***	314	6.620	1.342	8.2E-07	6.033	1.307	3.9E-06

$SE: Standard error

*Cell-count adjusted model was adjusted for age, sex, Framingham family structure and different cell counts

+Multivariable adjusted model was adjusted for age, sex, Framingham family structure, and additional risk factors including smoker, height, weight, systolic blood pressure, diastolic blood pressure, prevalent diabetes mellitus, prevalent myocardial infarction, prevalent heart failure and antihypertensive treatment.

Supplemental Table 2. Association of top CpG sites with both AF (combining prevalent and incident AF).

CpG site	Chr	Position[+]	Closest gene	Distance	Effect size	SE[*]	*P* value
cg21717959	6	163148762	***PARK2***	5'UTR	0.003	0.001	5.9E-06
cg24173182	17	1961286	***HIC1***	Exon 2	-0.013	0.003	7.1E-06
cg06690548	4	139162808	***SLC7A11***	Intron 1	-0.018	0.004	7.2E-06
cg14055835	19	55384515	***FCAR***	Promoter	0.020	0.004	7.6E-06
cg25451120	17	47287444	***GNGT2***	Intron 1	0.012	0.003	8.7E-06
cg01494454	12	25055214	***BCAT1***	Intron 1	-0.004	0.001	8.8E-06

[+]NCBI Genome Build 37
[*]SE: Standard error
